# A low serum uric acid concentration predicts a poor prognosis in adult patients with candidemia

**DOI:** 10.1515/med-2022-0511

**Published:** 2022-06-09

**Authors:** Yuqi Zhou, Wenjuan Li, Yubo Huang

**Affiliations:** Department of Pulmonary and Critical Care Medicine, The Third Affiliated Hospital, Sun Yat-Sen University, 600 Tianhe Road, Tianhe District, Guangzhou, Guangdong, 510630, P.R. China

**Keywords:** uric acid, cystatin C, candidemia, infection

## Abstract

This study aimed to determine the relation of serum uric acid (UA) level with outcomes in adults with candidemia. Medical records of patients with candidemia treated from 2014 to 2017 were retrospectively reviewed. Patients were age- and sex-matched with healthy control subjects. The associations of UA and cystatin C (CysC) levels with diagnosis and prognosis of candidemia were determined. Sixty-four patients with candidemia (13 females and 51 males; mean age 48.5 years) and 64 matched control subjects were included. The median UA level of patients with candidemia was 255 μmol/L (range, 158–395 μmol/L), and of healthy controls was 398 μmol/L (range, 345–450 μmol/L) (*P* < 0.001). The median CysC level of patients with candidemia was 1.07 mg/L (range, 0.89–1.59 mg/L), and of the healthy controls was 0.82 mg/L (range, 0.74–0.95 mg/L) (*P* < 0.001). Patients with a favorable prognosis had significantly higher serum UA levels than those with a poor prognosis (181 μmol/L vs 344 μmol/L; *P* = 0.001). It was indicated that the estimated OR for UA was significantly > 1 (*P* = 0.009), and the AUC was 0.734. In summary, a lower serum UA level is associated with a diagnosis of candidemia, and a poor outcome.

## 1 Introduction

Invasive candida infection is mostly seen in immune deficient and critically ill patients [1]. Candidemia is the presence of a *Candida* species in the bloodstream, and it is the fourth most common cause of nosocomial bloodstream infections [2]. It is associated with severe morbidity, and patients with catheter-related infections often have comorbid candidemia [1,2,3]. It can rarely be detected by a positive blood culture, and thus it is a difficult clinical decision to treat a presumed infection [1,2].

Uric acid (UA) is a natural product of the purine metabolic pathway [4]. Studies have suggested that UA is a strong peroxynitrite scavenger and natural antioxidant [5,6]. UA has been found to stimulate the maturation of dendritic (DC) cells in T cell responsive immune function [4,6,7]. Thus, an appropriate UA level may enhance the body’s ability to fight with infection. However, high UA levels stimulate the innate immune system and are associated with a variety of autoimmune disorders [8,9], while low UA levels are seen in critically ill patients [10,11,12]. The complete role of UA with respect to the immune system and infection remains poorly understood.

Prior studies have shown that UA and CysC both play a role in inflammatory processes, and exert immune-related effects against many inflammatory diseases, including sepsis and auto-immune diseases [13,14,15]. Studies have also shown that CysC deficiency leads to an enhanced activation of dendritic cells as antigen-presenting cells, both *in vivo* and *ex-vivo* [12,16]. Candidemia is an immune-related infectious disease, and immune cells such as B cells and T cells are important effectors and regulators of immune responses in candidemia [1]. Interestingly, study has also shown that elevated CysC is associated with increased rates of community-acquired sepsis [13]. Although the mechanism behind the association of lower serum UA and higher CysC levels with candidemia is unknown, experimental evidence suggests the role of CysC and UA in the pathogenesis of candidemia.

The purpose of this study was to examine UA levels in patients with candidemia to determine if UA level has diagnostic and/or prognostic value in these patients.

## 2 Materials and methods

### 2.1 Patients and control subjects

The records of patients with candidemia treated at our institution from January 2014 to December 2017 were retrospectively reviewed. Inclusion criteria were: (1) hospitalized patients whose blood cultures were positive for Candida and had infectious fever-like manifestations during hospitalization; (2) clinical diagnosed with Candidemia. A patient who tested positive for fungemia multiple times during the same hospitalization was defined as an episode of fungemia. Patients with inaccurate or missing data and medical records were excluded. Meanwhile, age- and sex-matched healthy individuals were selected from the same time period to serve as a control group. The age and sex of control subjects were matched with those of patients using the propensity score matching method. All patients with candidemia were followed-up for a minimum of 2 months after first positive blood culture result.

### 2.2 Definitions and measurements

Candidemia was defined as the culture isolation of a *Candida* spp. from at least one peripherally obtained blood specimen in a patient with clinical signs and symptoms of candidemia according to the 2016 Infectious Diseases Society of America Clinical Practice Guideline for the Management of Candidiasis [17].

Blood specimens were cultured for identification of *Candida* spp. using an automated broth microdilution system (MicroScan WalkAway 96 Plus, Siemens Inc., United States), according to the manufacturer’s instructions. Peripheral blood was also tested for white blood cell (WBC) count, neutrophils, platelet count, lactate dehydrogenase (LDH), 1-3-β-d-glucan (Fungus[1,2,3]-β-d-Glucan Test, Chromogenic Method, Genobio, China), C-reactive protein (CRP), UA, and cystatin C (CysC) levels. Comorbidities were recorded in all patients.

This study was approved by the institutional review board (IRB) of the Third Affiliated Hospital of Sun Yat-Sen University (No. [2020]-02-257-01), and written informed consent was waived by the IRB due to the retrospective nature of this study.

### 2.3 Biochemical assays

Serum UA and CysC concentrations were measured by a direct enzymatic method, as described in a previous publication [17], using a Clinical Analyzer 7180-ISE (Hitachi High-Technologies, Tokyo, Japan). The serum UA reference range for males was 210–430 μmol/L, and for females was 150–360 μmol/L. The serum CysC reference range for males and females was 0.55–1.55 mg/L.

### 2.4 Follow-up evaluations

Patients with candidemia were followed-up in our hospital, and divided into 2 groups; those with a poor outcome and those with a favorable outcome. A favorable outcome was defined as body temperature turned to normal after treatment and evidence of infection improvement within 2 weeks of the diagnosis. A poor outcome was defined as a sustained fever after treatment and evidence of ongoing infection or death within 2 months of the diagnosis. The favorable and poor outcomes were mutually exclusive.

### 2.5 Statistical analysis

Continuous variables were presented as median and interquartile range (IQR: Q1–Q3), and were compared using the Mann–Whitney *U* test. The Shapiro–Wilk test was used to test the normality of continuous variables. Categorical variables were presented as number and percentage, and were compared using the chi-square test. Logistic regression and receiver operating characteristic (ROC) curve analyses were performed to examine the diagnostic performance of UA and CysC levels for the infection and outcomes of candidemia. Results were reported as estimated odds ratios (OR), and sensitivity and specificity based on maximization of the Youden index. Statistical analyses were performed using SPSS version 25 software (IBM Corporation, Somers, New York). All tests were 2-tailed, and a value of *P* < 0.05 was considered to indicate statistical significance.

## 3 Results

### 3.1 Patient demographic and clinical features

Sixty-four patients were included in the study, 13 females and 51 males with a mean age of 48.5 years. Of the patients, there were 28 cases of *Candida albicans*, 12 cases of *Candida tropicalis*, 9 cases of *Candida glabrata* complex, 7 cases of *Candida parapsilosis* complex, 3 cases of *Rhodotorula rubra*, 2 cases of *Candida lusitaniae*, 2 cases of *Candida krusei*, and 1 case of *Saccharomyces cerevisiae*.

Of the patients, 57 had central venous catheterization, 44 had a urinary catheter, 27 received invasive mechanical ventilation, 16 received hemodialysis, 8 had received gastrointestinal surgery, 26 received parenteral nutrition, and 10 patients received an immunosuppressive therapy. All patients had been treated with intravenous broad-spectrum antibiotics.

Of the 64 patients with candidemia, 12 (18.8%) had complications of hematological malignancies, 15 (23.4%) had malignant solid tumors, 28 (43.8%) had pneumonia, 3 (4.7%) received a renal transplantation, 12 (18.8%) had renal failure, 6 (9.4%) had diabetes, 2 (3.1%) had liver failure, and 6 (9.4%) had peritonitis. 32 of the patients had unfavorable outcomes, and 12 of these patients died.

### 3.2 Comparison of serum UA and CysC levels between patients with candidemia and healthy control subjects

**Figure 1:**
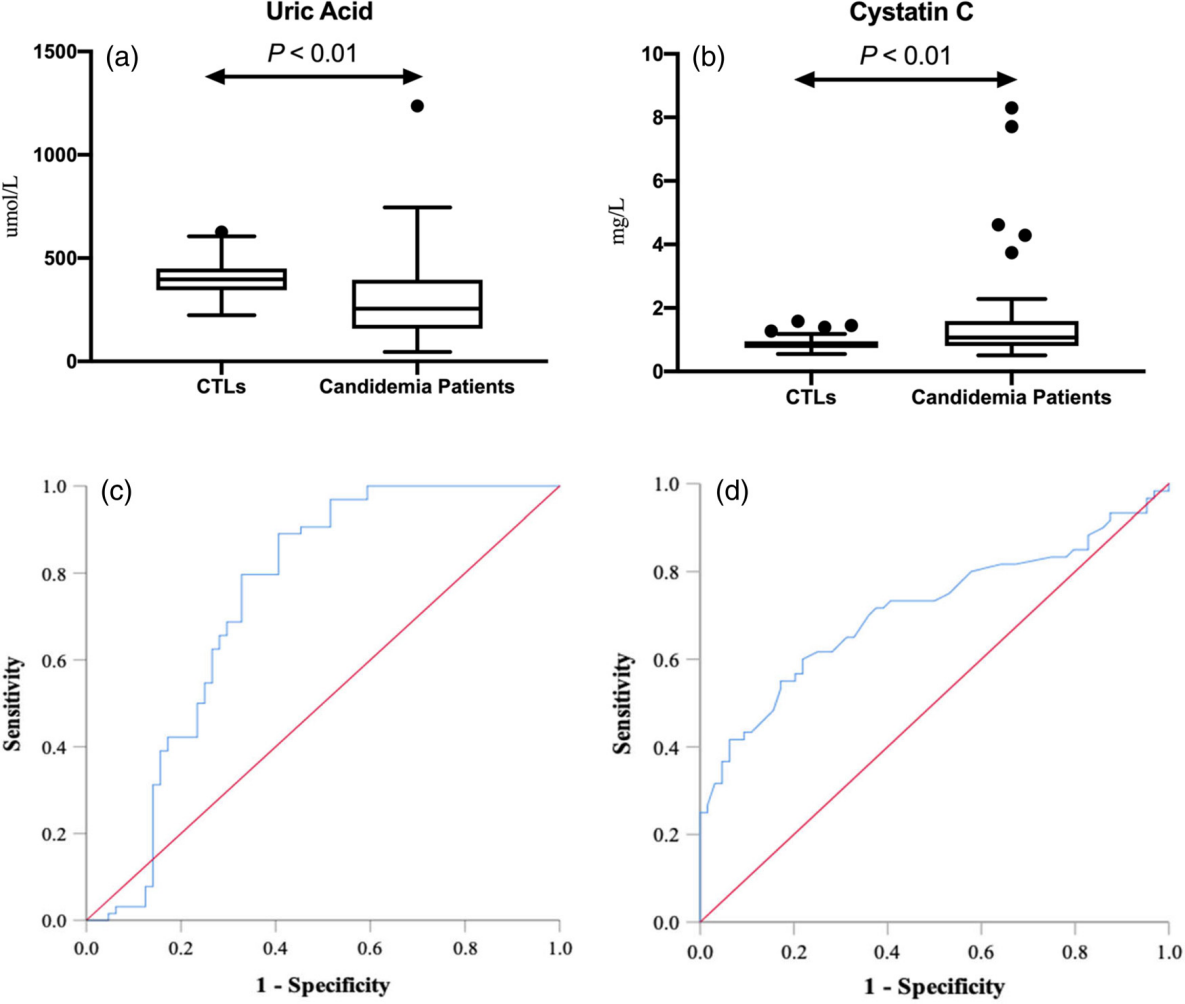
Box-whisker plot for UA (a) and cystatin C (b), and receiver operating characteristic curve for UA (c), and cystatin C (d) in patients with or without candidemia.

The median UA level of patients with candidemia was 255 μmol/L (range, 158–395 μmol/L) and the median level of healthy control subjects was 398 μmol/L (range, 345–450 μmol/L) (*P* < 0.001) (Table 1). As shown in Figure 1a, serum UA levels in patients with candidemia were significantly lower than that in healthy control subjects (*P* < 0.001). The median CysC level of patients with candidemia was 1.07 mg/L (range, 0.89–1.59 mg/L), and that of the healthy control subjects was 0.82 mg/L (range, 0.74–0.95 mg/L) (*P* < 0.001). CysC levels in patients with candidemia were significantly higher than that in healthy control subjects (Figure 1b).

**Table 1:** *Candida* species identified on culture and drug resistance

Antifungal drug	Resistance	*Candida albicans*	*Candida tropicalis*	*Candida glabrata*	*Candida kurou*	Others	Total
		(28 strains)	(12 strains)	(9 strains)	(2 strains)	(13 strains)	(64 strains)
5-Fluorine cytosine	Resistant	0	0	0	0	0	0
	Intermediate	0	0	0	1	0	1
Amphotericin B	Resistant	0	0	0	0	0	0
	Intermediate	0	0	0	0	0	0
Fluconazole	Resistant	3	5	0	1	0	9
	Intermediate	2	1	2	1	0	6
Itraconazole	Resistant	4	4	1	0	0	9
	Intermediate	4	3	2	1	0	10
Voriconazole	Resistant	3	5	0	0	0	8
	Intermediate	2	1	2	0	0	5

Intermediate indicates an intermediate level of resistance to the drug.

Results of the logistic regression and ROC analysis of the relations between UA and CysC level with candidemia are summarized in Table 2. The estimated OR of UA level was significantly <1, while the OR of CysC was >1 (both, *P* < 0.01), indicating that a low UA level or high CysC level were associated with a diagnosis of candidemia. The AUCs for both UA and CysC were >0.7, indicating moderate diagnostic performance (Figure 1c and d). For a diagnosis of candidemia, the suggestive cutoff value for UA was 292.15 μmol/L and that for CysC was 0.96 mg/L; both UA and CysC had comparatively better specificity than sensitivity.

**Table 2:** Serum uric acid and cystatin C concentrations of patients with candidemia and healthy control subjects

Variable	Candidemia patients	Matched control subjects	*P*
Number (female/male)	13/51	13/51	1.00
Age (years)	48.5 (30–60.8)	50 (31–63)	0.656
Uric acid (µmol/L)	255 (158–395)	398 (345–450)	<0.001
Cystatin C (mg/L)	1.07 (0.80–1.59)	0.82 (0.74–0.95)	<0.001

Age, uric acid, and cystatin C concentrations reported as median (IQR).

Differences between groups were evaluated with the Mann–Whitney *U* test.

### 3.3 A low serum UA level is associated with a poor prognosis in patients with candidemia

**Figure 2:**
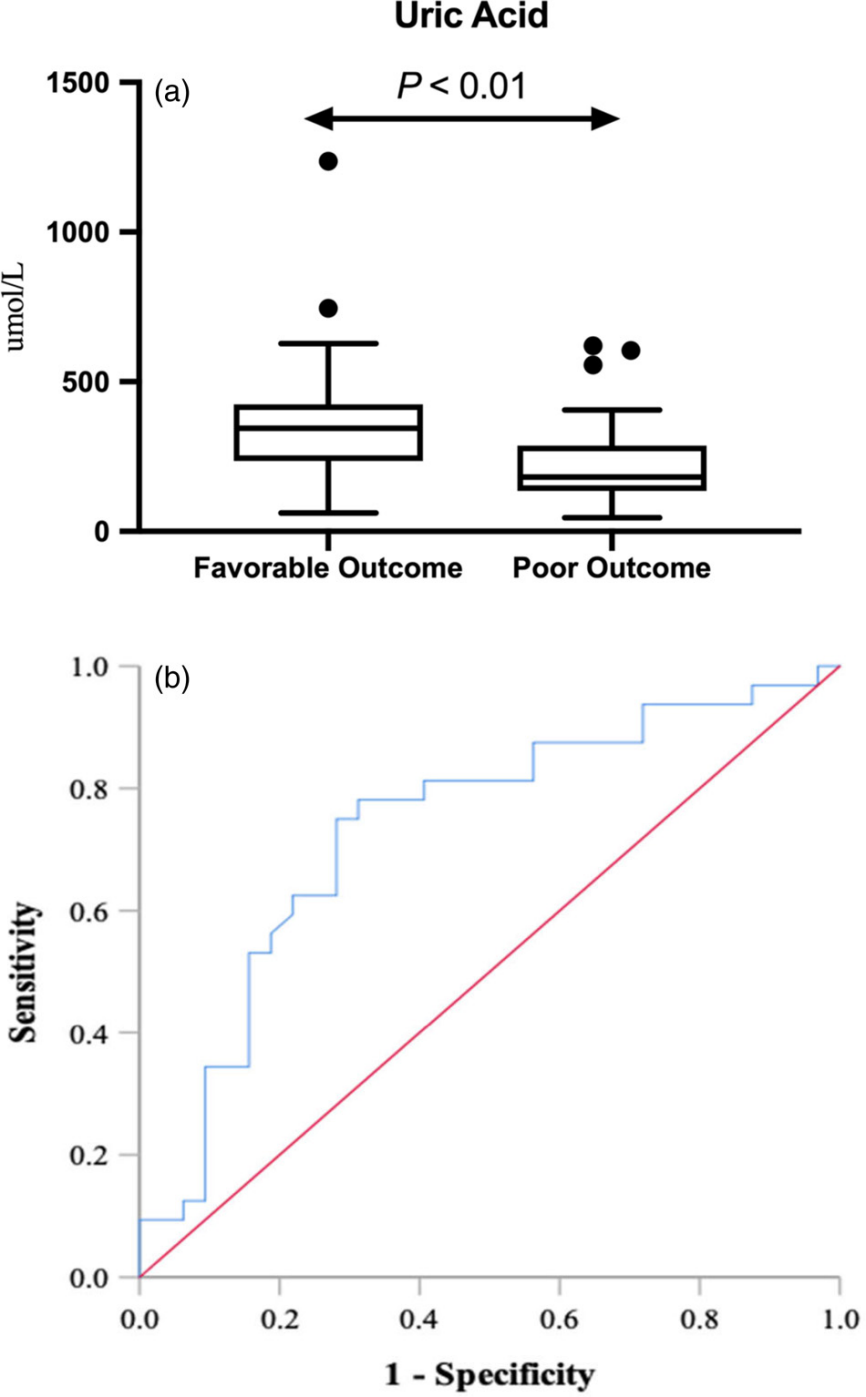
Box-whisker plot (a) and receiver operating characteristic curve (b) for UA in candidemia patients with different outcomes.

Of the 64 patients with candidemia, 32 cases had poor outcome, while the other 32 cases had favorable outcome. Patients with a favorable prognosis had significantly higher serum UA levels than those with a poor prognosis (181 μmol/L, range 135–287 μmol/L vs 344 μmol/L, range 236–424 μmol/L; *P* = 0.001) (Table 3 and Figure 2a). No significance was found in CysC level between patients with favorable prognosis (1.07 mg/L, range 0.51–2.28 mg/L) and poor prognosis (1.06 mg/L, range 0.56–8.30 mg/L) (*P* = 0.745).

**Table 3:** Logistic regression and ROC analyses of the relations of uric acid and cystatin C levels with candidemia

	Logistic regression							
Parameter	OR (95% CI)	*P*	AUC (95% CI)	*P*	Suggested cutoff	Sensitivity	Specificity	Youden index
Uric acid (μmol/L)	0.995 (0.992–0.998)	0.001	0.741 (0.650–0.832)	<0.001	292.15	0.59	0.89	0.48
Cystatin C (mg/L)	13.17 (3.31–52.42)	<0.001	0.713 (0.619–0.806)	<0.001	0.96	0.60	0.78	0.38

AUC, area under the receiver operating characteristic (ROC) curve; CI, confidence interval; OR, odds ratio.

The age- and sex-matched healthy control group was used as the reference.

Logistic regression and ROC analysis results are summarized in Table 4. For UA level, the estimated OR was significantly >1 (*P* = 0.009) (Figure 2b), the AUC was 0.734, and the suggestive cutoff value was 229.9 μmol/L; a medium to high sensitivity and specificity were observed. These results indicate that a low serum UA level is a predictor of a poor prognosis in patients with candidemia.

**Table 4:** Blood test results of patients with candidemia based on outcome

Blood test	Poor outcome (*n* = 32)	Favorable outcome (*n* = 32)	Total patients	*P*
	Number/Median (IQR)	Number/Median (IQR)	Median (IQR)	
WBC (×9/L)	32/8.7 (4.3–16.4)	32/7.8 (5.0–11.0)	8.5 (4.7–13.6)	0.591
NEUT (×9/L)	32/7.4 (3.6–14.2)	32/6.1 (3.6–8.2)	6.2 (3.6–10.1)	0.365
PLT (×9/L)	32/115 (31–219)	32/180 (101–248)	158 (50.3–238.5)	0.134
LDH (U/L)	32/285 (237–643)	32/274 (210.5–318.5)	279.5 (227.3–462.8)	0.368
PCT (ng/mL)	21/1.43 (0.42–5.71)	19/0.5 (0.38–6.33)	1.2 (0.4–6.1)	0.655
1-3-β-d Glucan (pg/mL)	16/24.4 (9.2–231.1)	10/40.4 (9.7–86.0)	28.6 (9.7–110.3)	0.812
CRP (mg/L)	18/69.4 (31.5–96.9)	16/66.5 (24.6–154.8)	69.4 (31.5–104.8)	0.730
UA (μmol/L)	32/181 (135–287)*	32/344 (236–424)*	255 (158–395)	0.001*
Cystatin C (mg/L)	28/1.08 (0.83–1.51)	32/1.05 (0.79–1.62)	1.07 (0.80–1.59)	0.773

CRP, C reactive protein; IQR; interquartile range; LDH, lactate dehydrogenase; NEUT, neutrophils; PCT, procalcitonin; PLT, platelets; UA, uric acid; WBC, white blood cells.

*P < 0.01.

## 4 Discussion

In the present study, we investigated the relations of serum UA and CysC levels with candidemia, and to the best of our knowledge this was the first study to examine these relations. The results demonstrated that patients with candidemia had significantly lower levels of UA and higher levels of CysC than healthy control subjects, and that a low UA level was associated with a poor prognosis of patients with candidemia.

Our results showed that serum UA level was associated with disease prognosis; patients with worse outcomes, who may have more inflammatory oxidative injury, had significantly lower serum UA levels. UA is a potentially important contributor to the innate immune response to infection, and may provide a target for adjunct therapies [18,19]. The low level of UA has been reported to be an important contributor to inflammatory cytokine secretion, and dendritic cell and T cell responses that can affect priming of the immune system *in vivo* [20]. UA has also been shown to promote an acute inflammatory response in mice [21]. Our results suggest that candidemia patients with lower UA levels had worse outcomes; a UA cutoff value of 228.8 µmol/L had a sensitivity of 0.7812 and a specificity of 0.6875 (Youden index = 0.4688) for a poor outcome.

We speculate that lower serum UA levels in patients with candidemia may contribute to an imbalance in the immune system, resulting in less protection against inflammatory oxidative damage and immune cell deficiency. In our opinion, higher levels of CysC may cause a greater inflammatory response in patients with candidemia.

There are limitations to this study that should be considered. This was a retrospective study with relatively small sample size. We did not re-test UA and CysC levels in patients who had recovered. We did not examine UA and CysC levels in light of other risk factors for candidemia, such as neutropenic diseases, diabetes, renal failure, central venous catheter placement, renal replacement therapy, endotracheal intubation, and urinary catheters [22]. We did not examine potential underlying mechanisms, or the impact of immune status or antioxidant status on outcomes. Although *Candida* spp. were identified by using an automated broth microdilution system (MicroScan), we did not use a conventional method, such as cornmeal tween 80 agar technique to support the results. We did not have another independent dataset of candidemia patients to verify the conclusions from this study.

The results of this study indicate that a low UA level may be diagnostic of candidemia, and is associated with worse outcomes of patients with candidemia. Conversely, an elevated CysC level is associated with a diagnosis of candidemia. These data may assist in the diagnosis and treatment of patients with candidemia.
